# Assessment of global histone acetylation in pediatric and adolescent obesity: Correlations with SIRT1 expression and metabolic-inflammatory profiles

**DOI:** 10.1371/journal.pone.0293217

**Published:** 2023-10-20

**Authors:** Nima Taghizadeh, Soha Mohammadi, Zeynab yousefi, Pegah Golpour, Alemeh Taheri, Mohammad Hasan Maleki, Mitra Nourbakhsh, Mona Nourbakhsh, Maryam Razzaghy Azar

**Affiliations:** 1 Metabolic Disorders Research Center, Endocrinology and Metabolism Molecular-Cellular Sciences Institute, Tehran University of Medical Sciences, Tehran, Iran; 2 Endocrinology and Metabolism Research Center, Endocrinology and Metabolism Clinical Sciences Institute, Tehran University of Medical Sciences, Tehran, Iran; 3 Department of Clinical Biochemistry, Faculty of Medical Sciences, Tarbiat Modares University, Tehran, Iran; 4 Department of Biochemistry, School of Medicine, Shahid Sadoughi University of Medical Sciences, Yazd, Iran; 5 Department of Biochemistry, School of Medicine, Iran University of Medical Sciences, Tehran, Iran; 6 Department of Biochemistry, School of Medicine, Shiraz University of Medical Sciences, Shiraz, Iran; 7 Finetech in Medicine Research Center, Iran University of Medical Sciences, Tehran, Iran; 8 Hazrat Aliasghar Children Hospital, School of Medicine, Iran University of Medical Sciences, Tehran, Iran; Kerman University of Medical Sciences Physiology Research Center, ISLAMIC REPUBLIC OF IRAN

## Abstract

**Background:**

Epigenetic modifications, particularly histone acetylation-deacetylation and its related enzymes, such as sirtuin 1 (SIRT1) deacetylase, may have substantial roles in the pathogenesis of obesity and its associated health issues. This study aimed to evaluate global histone acetylation status and SIRT1 gene expression in children and adolescents with obesity and their association with metabolic and anthropometric parameters.

**Methods:**

This study included 60 children and adolescents, 30 with obesity and 30 normal-weight. The evaluation consisted of the analysis of global histone acetylation levels and the expression of the *SIRT1* gene in peripheral blood mononuclear cells, by specific antibody and real-time PCR, respectively. Additionally, insulin, fasting plasma glucose, lipid profile and tumor necrosis factor α (TNF-α) levels were measured. Insulin resistance was assessed using the homeostasis model assessment of insulin resistance (HOMA-IR). Metabolic syndrome was determined based on the diagnostic criteria established by IDF.

**Results:**

Individuals with obesity, particularly those with insulin resistance, had significantly higher histone acetylation levels compared to control group. Histone acetylation was positively correlated with obesity indices, TNF-α, insulin, and HOMA-IR. Additionally, a significant decrease in *SIRT1* gene expression was found among obese individuals, which was negatively correlated with the histone acetylation level. Furthermore, *SIRT1* expression levels showed a negative correlation with various anthropometric and metabolic parameters.

**Conclusion:**

Histone acetylation was enhanced in children and adolescents with obesity, potentially resulting from down-regulation of SIRT1, and could play a role in the obesity-associated metabolic abnormalities and insulin resistance. Targeting global histone acetylation modulation might be considered as an epigenetic approach for early obesity management.

## Introduction

Obesity is a significant public health concern that has been associated with an increased risk of various metabolic and cardiovascular diseases [1]. Obesity puts individuals at risk for diseases such as insulin resistance, type 2 diabetes, hypertension, non-alcoholic fatty liver disease (NAFLD), and cancer [2, 3]. Obesity is also common in children and is increasingly prevalent [4], leading to a higher risk of developing metabolic syndrome and vascular diseases. Additionally, obese children and adolescents are at greater risk of obesity and its complications in adulthood, including higher mortality rates than children with normal weight [5, 6].

In addition to lifestyle factors, genetic and epigenetic factors have also been linked to the development of obesity and its associated complications [7]. Among these epigenetic factors, histone post-translational modifications, specifically acetylation and methylation, play a crucial role in gene expression control mechanisms and significantly impact metabolic and hormonal changes associated with obesity [8]. They also have been found to contribute to metabolic outcomes of obesity such as insulin resistance, dysfunction in gluconeogenesis, and impairment in lipogenesis [9].

Histones are small, positively charged proteins, involved in packaging of DNA into nucleosomes. There are five types of histones in humans: H1, H2A, H2B, H3, and H4. While H2A, H2B, H3, and H4 form the core unit of chromatin, H1 acts as a linker protein. The interaction between histones and DNA stabilizes the nucleosome, but hinders transcription, replication, and DNA repair. Post-translational modifications like acetylation weaken the histone-DNA interaction, destabilizing the nucleosome [10].

Histone acetyltransferase enzymes acetylate histones, weakening their connection to DNA and facilitating transcription, while histone deacetylase enzymes remove acetyl groups and strengthen the connection between histones and DNA, leading to reduced gene expression. Enzymes such as CBP/P300, GCN5, and PCAF are important histone acetyltransferases, and important deacetylases include HDAC1 and sirtuins [8]. Of these, sirtuins have received significant attention due to their crucial regulatory role in various cellular processes, particularly carbohydrate and lipid metabolism [11]. Sirtuin 1 (SIRT1), which is an important histone deacetylase and is predominantly located in the nucleus, plays a crucial role in protecting cells against oxidative stress and regulating metabolic pathways, particularly in adipose tissue [12, 13].

In adipose cells, SIRT1 acts as an inhibitor of adipogenesis, and also plays a major role in regulating the expression and secretion of adiponectin, an anti-inflammatory hormone that is decreased in obesity and type 2 diabetes [13, 14]. SIRT1 is expressed in PBMCs [15], and increasing in vitro and in vivo evidence indicates that its expression is reduced during the acute inflammatory response and associated conditions, whereas boosting SIRT1 expression, for example by certain drugs and molecules has an anti-inflammatory effect [16].

Despite the fundamental relationship between histone acetylation and metabolic homeostasis and the extensive research on obesity, the status of global histone acetylation and its association with the expression of controlling enzyme genes in childhood obesity have not been explored. Therefore, this study aimed to investigate changes in global histone acetylation in children and adolescents and its relationship with inflammatory markers and the expression of the *SIRT1* gene. The investigation will help to identify the role of histone acetylation in the development of obesity in children and help to prevent the progression of associated complications.

## Materials and methods

### Subjects

The study involved 60 participants, aged between 8 and 18, consisting of 26 girls and 34 boys, with a mean age of 11.09±2.31 years. The participants were recruited during 2022 from the outpatient Endocrinology Clinic of Hazrat Aliasghar Children Hospital and went through a complete clinical evaluation and 30 subjects with obesity and 30 healthy normal-weight control subjects of the same age and gender were carefully selected. Age of 8–18 years, both males and females, and BMI higher than the 95th percentile for their age and sex, based on the clinical growth charts developed by Centers for Disease Control and Prevention (CDC), were considered as the inclusion criteria for the obesity group. Similar age range and genders, together with BMI falling within the normal range (5th to 85th percentile) were considered as the inclusion criteria for the normal-weight control group. Exclusion criteria comprised of obesity caused by endocrine diseases such as hypothyroidism and Cushing’s syndrome, presence of any acute or chronic illnesses, and intake of medications. Control subjects were healthy and met the aforementioned exclusion criteria.

The family and health history were taken from all participants, and they underwent a thorough clinical examination. Height and weight were recorded, and the body mass index (BMI) was calculated as weight/height^2. Hip circumference (HC) and waist circumference (WC) were measured with a tape measure, the WC/HC ratio was calculated, and the WC percentiles were determined [17]. The z-scores for weight and height were calculated using the CDC weight-for-age and height-for-age growth charts, which are stratified by sex. Diastolic blood pressure (DBP) and systolic blood pressure (SBP) were also recorded.

All participants or their parents provided written informed consent. The study was approved by the ethics committee of the Iran University of Medical Sciences (ethics code IR.IUMS.REC.1399.115) and the Endocrinology and Metabolism Research Institute, Tehran University of Medical Sciences (ethics code IR.TUMS.EMRI.REC.1401.061), and followed the guidelines of the Helsinki Declaration and the Iranian Ministry of Health and Medical Education.

### Sample collection and PBMC separation

Blood samples were drawn from the subjects after at least 12 hours of overnight fasting and collected in plain and EDTA-containing tubes to separate serum and peripheral blood mononuclear cells (PBMCs), respectively. PBMCs were separated using Lympholyte-H density gradient separation medium (Cedarlane Laboratories, Canada) to produce a density gradient, followed by washing with phosphate-buffered saline and centrifugation. The cells were counted with a hemocytometer using trypan blue dye. Serum samples, as well as PBMCs, were aliquoted and stored at -80°C for future experiments.

### Biochemical tests

The lipid profile, including triglycerides (TG), total cholesterol (TC), low-density lipoprotein cholesterol (LDL-C), and high-density lipoprotein cholesterol (HDL-C), were measured using direct enzymatic methods by commercial kits (Pars Peyvand, Iran). Fasting plasma glucose (FPG) was also measured by an enzymatic method (Pars Peyvand, Iran). TNF-α and insulin were measured using enzyme-linked immunosorbent assay (ELISA) kits (Cusabio, China, and Monobind, USA, respectively). Using the concentrations of insulin and glucose, the Homeostatic Model Assessment of Insulin Resistance (HOMA-IR) was calculated as serum insulin (μIU/ml) x FPG (mg/dl)/405 to evaluate insulin resistance [18]. The cutoff level of 3.16 was used to discriminate the subjects with insulin resistance [19].

### Determination of metabolic syndrome

The guideline of the International Diabetes Federation, was used for the determination of metabolic syndrome (MetS), considering waist circumference (WC) higher than the 90th percentile indicating abdominal obesity, and the presence of two or more of the metabolic characteristics, including high levels of TG, glucose, DBP, or SBP, or lower than normal levels of HDL-C [20].

### Gene expression analysis

Cells were lyzed and their RNA content was isolated using the Rapid Blood RNA Isolation Kit (BioBasic, Canada). The quality and quantity of the extracted RNA were determined using a nanodrop, and the complementary DNA (cDNA) was synthesized using the High-Capacity cDNA Reverse Transcription Kit (Applied Biosystems, USA). Real-time PCR was performed to estimate the expression of the *SIRT1* gene using SYBR Green master mix (Ampliqon, Denmark). The expression of the housekeeping gene *ACTB* (encoding β-actin) was determined and used to normalize the expression of *SIRT1*. The primers used were: *ACTB*: 5´- GCAAGCAGGAGTATGACGAG (forward), 5´- CAAATAAAGCCATGCCAATC (reverse); *SIRT1*: 5´-TGCGGGAATCCAAAGGATAA (forward), 5´-CAGGCAAGATGCTGTTGCA (reverse). A standard curve was created using pooled cDNA samples diluted in serial concentrations to determine the PCR efficiency. The level of gene expression was estimated by the 2^-ΔCt^ method, using the formula: ΔCt = Ct (ACTB)-Ct (SIRT1).

### Histone H3 acetylation assay

Histone H3 acetylation was assessed using an assay kit (Abcam, UK) and a specific antibody against acetylated H3 histone, following the manufacturer’s protocol. Briefly, 10^6^ cells from each PBMC sample were centrifuged at 1000 rpm for 5 min and washed with 10 mL PBS and centrifugation at 1000 rpm for 5 min. Lysis buffer (10 μL) was added to the cells and incubated on ice for 5 minutes with occasional vortexing. To prepare histone extracts, 3 volumes of the extraction buffer/glycerol solution was mixed with the cell lysate and incubated on ice for 5 min. After removal of debris by centrifugation at 12000 rpm for 5 min, precipitation was performed by the addition of 100% trichloroacetic acid (TCA) at 1:4 ratio, and incubation on ice for 30 min. The precipitate was collected by centrifugation at 12000 rpm for 10 min at 4°C, and was mixed with 1 mL of acetone containing 0.1% HCl and incubated for 1 min. The pellet was collected by centrifugation and the air-dried precipitate was dissolved in 10 μL distilled water and the protein concentration was measured.

For each sample, 200 ng/μL protein was dispensed into each well of a 98-well plate strip and allowed to dry at 37°C for 90 min. After blocking with 150 μL of blocking buffer and washing three times, 50 μL capture antibody was added to each well and incubated at room temperature for 60 min on a shaker. The cells were washed four times and then the secondary antibody (150 μL) was added to the wells, and after washing, color was produced by 100 μL of developing solution and incubation for 10 min away from light, followed by the addition of 50 μL stop solution. The absorbance was detected by a microplate reader at 450 nm. Acetylation was calculated by subtracting the blank OD from the sample OD.

### Statistical analyses

The collected data were analyzed using IBM SPSS version 24 (SPSS Inc., Chicago, IL, USA) and MedCalc version 18.9.10 (Ostend, Belgium). The normal distribution of the variables was assessed using the Kolmogorov-Smirnov test. Variables that showed normal distribution were analyzed using the independent sample t-test and are presented as mean ± standard deviation (SD). Variables that did not have a normal distribution were evaluated using the Mann-Whitney U test and presented as median (interquartile range). The relationship between variables was examined using Pearson or Spearman correlation analysis based on the parametric or non-parametric distribution of data, respectively. A *P* value lower than 0.05 was considered statistically significant.

## Results

The studied subjects’ anthropometric and biochemical characteristics are presented in Table 1. The two groups were matched and no significant variations were observed in age and gender between the two groups under investigation. Significant differences were observed between the case and the control groups in weight, weight z-score, BMI, BMI z-score, WC, hip circumference, WHR, systolic and diastolic blood pressure. The group with obesity exhibited higher values in all of these variables (Table 1).

**Table 1:** Demographic and biochemical characteristics of study population.

Characteristics	Participants		*P* value
	Obese (n = 30)	Normal (n = 30)	
**Age (years)**	11.09 ± 2.29	11.10 ± 1.99	0.97
**Gender (male/female)**	19 (63.33%)/11 (36.67%)	15 (50%)/15 (50%)	0.30
**Height (cm)**	**146.98 ± 7.76**	**140.32 ± 10.91**	**<0.001**
**Height z-score**	**0.46 ± 1.23**	**-0.65 ± 0.95**	**<0.001**
**Weight (kg)**	**65.14 ± 15.37**	**35.50 ± 7.08**	**<0.001**
**Weight z-score**	**2.27 ± 0.60**	**-0.37 ± 0.94**	**<0.001**
**BMI (kg/m2)**	**29.81 ± 4.59**	**17.84 ± 1.65**	**<0.001**
**BMI z-score**	**2.30 (2.17–2.50)**	**0.29 (-0.80–0.80)**	**<0.001**
**Waist circumference (cm)**	**94.20 ± 11.22**	**67.57 ± 8.16**	**<0.001**
**Hip circumference (cm)**	**100.30 ± 10.67**	**78.16 ± 7.25**	**<0.001**
**WHR**	**0.93 ± 0.05**	**0.86 ± 0.05**	**<0.001**
**SBP (mmHg)**	**127.17 ± 15.72**	**112 ± 10.76**	**<0.001**
**DBP (mmHg)**	**81.47 ± 10.07**	**72.57 ± 7.81**	**<0.001**
**Triglyceride(mg/dl)**	**107.00 (76.25–178.50)**	**76.50 (63.50–93.75)**	**<0.001**
**Cholesterol (mg/dl)**	159.66 ± 26.02	152.26 ± 27.89	0.29
**LDL-C (mg/dl)**	82.00 ± 16.68	74.56 ± 13.40	0.06
**HDL-C (mg/dl)**	**46.83 ± 7.84**	**52.73 ± 13.21**	**0.04**
**FPG (mg/dl)**	**93.76 ± 4.93**	**86.96 ± 6.20**	**<0.001**
**Insulin(μIU/mL)**	**11.95 (9.40–19.87)**	**5.4 (4.58–9.87)**	**<0.001**
**HOMA-IR**	**2.73 (2.15–4.56)**	**1.10 (0.88–2.15)**	**<0.001**
**TNF-α (pg/ml)**	**30.81 ± 5.00**	**22.30 ± 2.63**	**<0.001**

The data are presented as mean *±* standard deviation, numbers (proportions) or median (25 percentiles- 75 percentiles). BMI: Body mass index; WHR: Waist to Hip Ratio; SBP: Systolic blood pressure; DBP: Diastolic blood pressure; LDL-C: Low density lipoprotein cholesterol; HDL-C: High density lipoprotein cholesterol; FBG: Fasting plasma glucose; HOMA-IR: Homeostatic Model Assessment of Insulin Resistance; TNF- α: Tumor necrosis factor alpha.

Regarding the lipid profile, triglyceride levels were significantly elevated in the group with obesity compared to the control group. Additionally, HDL-C levels were lower in the case group, while there were no significant differences in total cholesterol and LDL-C levels between the two groups, which might be due to the young age of the studied subjects and the need for long-term effects of obesity to be reflected on LDL-C and cholesterol levels. In terms of other biochemical variables, the case group showed elevated levels of FPG, insulin, and the HOMA-IR index, indicating increased insulin resistance compared to the normal group, which suggests potential metabolic dysregulation in individuals with obesity. Additionally, TNF-α levels were significantly elevated in the group with obesity compared to the normal group (Table 1).

**Fig 1:**
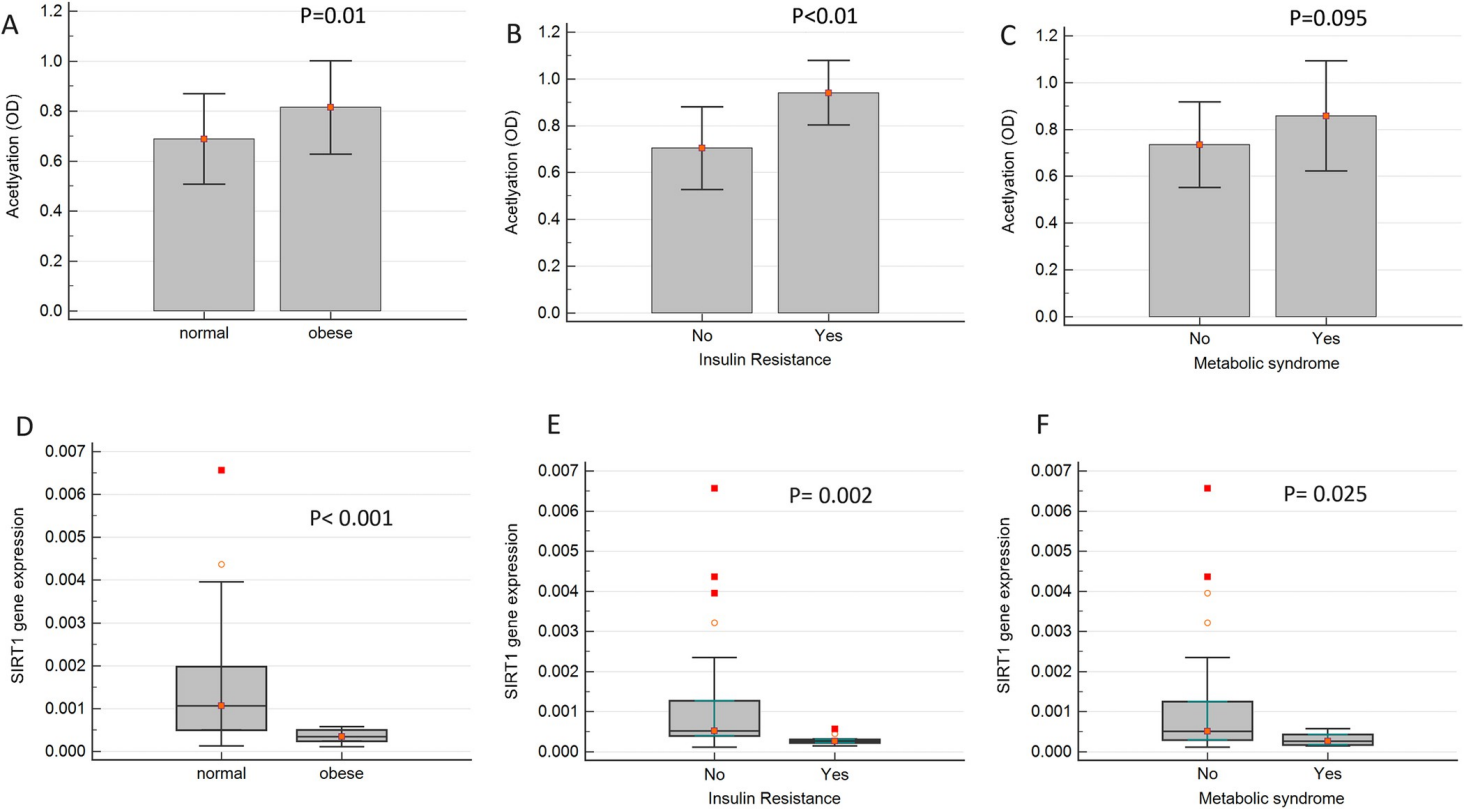
The levels of global histone acetylation. Histone acetylation in the PBMCs, (A) in children with obesity compared to normal-weight children, (B) in children with insulin resistance compared with those without insulin resistance, and (C) in children with metabolic syndrome compared with those without metabolic syndrome (C). SIRT1 gene expression levels in the PBMCs of (D) children with obesity compared to normal-weight children, (E) children with insulin resistance compared with those without insulin resistance, and (F) children with metabolic syndrome compared with those without metabolic syndrome. *P<0.05.

The study revealed that histone acetylation status was higher in the obese group compared to the control group (Fig 1A). Out of the studied participants, 20% had insulin resistance, and 13.33% were diagnosed with MetS, and the acetylation levels were compared in these two groups, separately. As it is shown in Fig 1B, the histone acetylation levels were significantly higher in the individuals with insulin resistance compared with the subjects with obesity who did not have insulin resistance. Children and adolescents with MetS also showed higher acetylation compared to those without MetS; however, the difference was not statistically significant (*P* value = 0.095) (Fig 1C).

**Fig 2:**
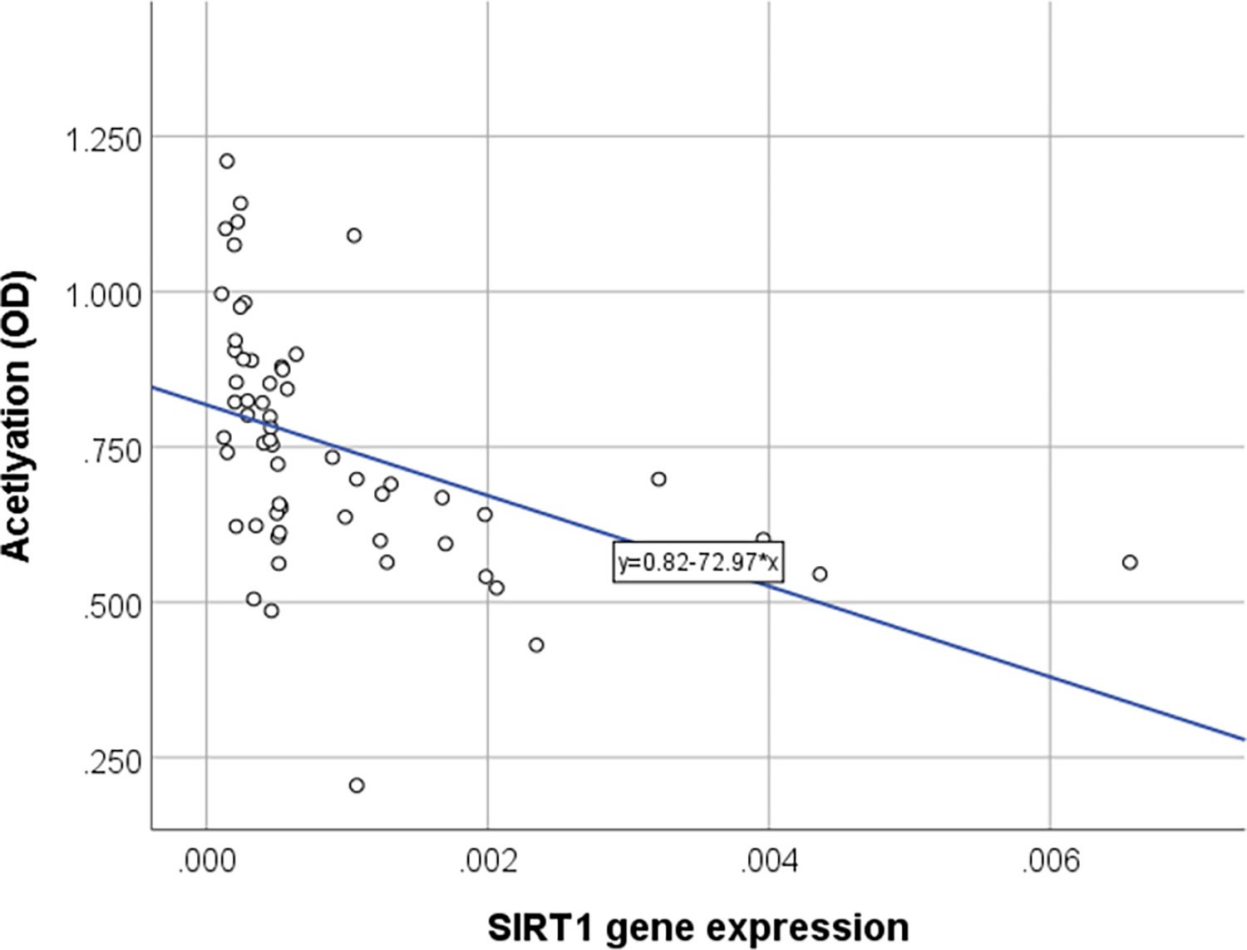
The correlation between histone acetylation level and the expression of SIRT1 gene.

We also examined the expression of the *SIRT1* gene and found it to be significantly lower in individuals with obesity compared with normal-weight children, as well as those with insulin resistance and MetS, compared with the children with obesity who did not present with insulin resistance or MetS (Fig 1D–1F). The study identified a significant negative correlation between histone acetylation and *SIRT1* gene expression (coefficient of rank correlation (rho) = -0.65, *P* = < 0.001) (Fig 2).

While age and height did not significantly correlate with *SIRT1* gene expression, the obesity characteristics including, weight, weight z-score, BMI, BMI z-score, waist circumference, and waist-to-hip ratio were notably related to *SIRT1* expression. Metabolic markers such as triglyceride, cholesterol, HDL-C, LDL-C, and FBG did not significantly correlate with *SIRT1* gene expression. However, notable negative correlations were observed between *SIRT1* gene expression and insulin levels, the HOMA-IR index, and TNF-α levels which were found to be statistically significant (Table 2).

**Table 2:** Correlations of SIRT 1 gene expression and histone acetylation with other variables.

Variables	SIRT 1 gene expression		Acetylation (OD)	
	R	*P*	R	*P*
**Age (years)**	-0.016	0.90	0.063	0.63
**Height (cm)**	-0.190	0.15	0.052	0.69
**Height z-score**	-0.224	0.86	0.085	0.52
**Weight (kg)**	**-0.448**	**<0.001**	**0.276**	**0.03**
**Weight z-score**	**-0.405**	**<0.001**	0.174	0.18
**BMI (kg/m2)**	**-0.400**	**<0.001**	**0.311**	**0.01**
**BMI z-score**	**-0.376**	**<0.001**	0.245	0.06
**Waist circumference (cm)**	**-0.373**	**<0.001**	**0.270**	**0.04**
**Waist to hip ratio**	**-0.268**	**0.04**	0.227	0.08
**SBP (mmHg)**	-0.170	0.19	0.153	0.24
**DBP (mmHg)**	-0.197	0.13	0.201	0.12
**Triglyceride(mg/dl)**	-0.07	0.59	0.089	0.49
**Cholesterol (mg/dl)**	-0.038	0.77	0.010	0.93
**HDL-C (mg/dl)**	0.09	0.50	-0.159	0.22
**LDL-C (mg/dl)**	-0.022	0.86	0.047	0.72
**FBG (mg/dl)**	-0.111	0.40	0.179	0.17
**Insulin (μIU/mL)**	**-0.375**	**<0.001**	**0.424**	**<0.001**
**HOMA Index**	**-0.389**	**<0.001**	**0.436**	**<0.001**
**TNF-α (pg/ml)**	**-0.399**	**<0.001**	**0.358**	**<0.001**

BMI: Body mass index; WHR: Waist to Hip Ratio; SBP: Systolic blood pressure; DBP: Diastolic blood pressure; LDL-C: Low density lipoprotein cholesterol; HDL-C: High density lipoprotein cholesterol; FBG: Fasting plasma glucose; HOMA-IR: Homeostatic Model Assessment of Insulin Resistance; TNF- α: Tumor necrosis factor alpha.

Regarding histone acetylation levels, age, and height were not significantly correlated, while weight, BMI, and waist circumference showed significant positive correlation. Among the MetS components, acetylation levels did not have significant associations with blood pressure measurements, and no significant relationships were found between acetylation and the levels of TG, TC, HDL-C, and LDL-C, or FPG. However, strong positive correlations were observed between acetylation and insulin levels and the HOMA-IR. Acetylation also showed a strong positive correlation with TNF-α levels (Table 2).

## Discussion

The etiology of obesity is multifactorial, resulting from the intricate interplay of lifestyle factors, environmental influences, and genetic and epigenetic mechanisms [21]. Perturbation in the expression of various genes has been demonstrated to be associated with the pathogenesis of obesity [22]. In the present study we demonstrated for the first time that histone acetylation state is altered in children and adolescents with obesity and that these subjects encounter higher acetylation in their histone proteins compared to normal-weight healthy children, indicating that this epigenetic alteration occurs in obesity at young age. We also found that there exists a potential correlation between histone acetylation levels and obesity-related variables such as weight, BMI, BMI z-score, waist circumference, and waist-to-hip ratio, thereby indicating an alleged involvement of acetylation in the regulation of body weight, body fat distribution, and BMI. The significant alteration of histone acetylation in children and adolescents with obesity highlights that the fundamental epigenetics changes in obesity occur at an early age.

Histone H3 acetylation plays a crucial role in the development and progression of obesity by regulating active gene transcription and is implicated in the metabolic dysfunction associated with obesity [23]. It has been shown in previous research that the acetylation of histone H3 leads to nucleosome disassembly, while acetylation of H4 alone promotes unwrapping of the DNA ends, but does not increase disassembly compared to unmodified histones [24]. Enzymes participating in energy metabolism also undergo acetylation, including those related to glycolysis, glucose oxidation, TCA cycle, electron transport chain, and fatty acid ß-oxidation [25, 26]. The acetylation process is sensitive to changes in nutrient status, such as high-fat feeding or caloric restriction [27, 28].

Schwer and colleagues conducted a comprehensive analysis of acetylation of mitochondrial enzymes in mice models of calorie restriction and found that calorie restriction significantly reduced the acetylation of mitochondrial proteins, specifically in brown adipose tissue [27]. This observation suggests a potential relationship between acetylation, mitochondrial metabolism, and the complicated energy balance mechanisms involved in obesity pathophysiology. Hirschey et al. also studied hepatic mitochondrial protein acetylation and SIRT3 activity in mice model of obesity and reported hyperacetylation of these proteins and suppression of SIRT3 enzyme in response to high fat diet feeding. So they suggested loss of SIRT3 and enhanced mitochondrial protein acetylation as contributors to the development of MetS [29]. In another study, Shepard et al. performed a proteomics analysis on the liver proteins of ethanol-fed rats and demonstrated hyperacetylation of several proteins, which may be responsible for the development of liver injury [30]. Consistent with our findings, these outcomes suggest that dysregulated acetylation processes play a crucial role in the development and progression of obesity and related metabolic disorders.

McGee et al. conducted a study revealing that following exercise, there is a widespread increase in H3 lysine acetylation in human muscle biopsies. The observed escalation in acetylation levels post-exercise suggests a potential activation of genes associated with metabolic pathways, thereby facilitating weight management and promoting the utilization of adipose stores [31].

The current study revealed a significant downregulation of *SIRT1* gene expression in the peripheral blood mononuclear cells (PBMCs) of obese children and adolescents compared to individuals with normal weight. These results are consistent with previous studies demonstrating lower *SIRT1* gene expression in adipose tissues of individuals with obesity, as well as lower blood levels of SIRT1 in patients with diabetes [32–37]. It has also been shown that SIRT1 mRNA levels significantly increases in subcutaneous adipose tissue in response to weight loss [38]. We also showed that the downregulation of *SIRT1* gene was correlated with the augmentation of histone acetylation, which suggests a causative relationship between histone epigenetics alterations and suppression of SIRT1. The *SIRT1* gene, encoding an NAD^+^-dependent histone deacetylase, is pivotal in obesity, regulating energy homeostasis, glucose metabolism, and lipid utilization [39]. SIRT1 also plays a role in deacetylating SIRT3 within the mitochondria. SIRT3 is an enzyme whose hyperacetylation is observed in aged and obese mice, and SIRT1 activity increases its stability. Notably, when SIRT3 is rendered acetylation-defective, it demonstrates the capacity to reverse metabolic abnormalities resulting from a high-fat diet in mice. This highlights the pivotal role of SIRT1-mediated deacetylation in preventing obesity-related metabolic syndrome [40]. Furthermore, Vázquez et al. demonstrated that early-onset over-nutrition leads to the suppression of SIRT1, which in turn, enables SIRT1 to exert epigenetic regulation over genes involved in the onset of puberty [41].

Furthermore, our research discovered that individuals with insulin resistance and metabolic syndrome have notably lower levels of *SIRT1* gene expression compared to those who do not have these conditions. We found a significant inverse relationship between the expression of the *SIRT1* gene and insulin levels, as well as the HOMA-IR index, which implies that SIRT1 plays a substantial role in regulating insulin resistance. The significant positive correlation between histone acetylation levels and insulin resistance indices suggests that long-term induction of insulin resistance in obesity and in cases with low *SIRT1* expression might be mediated by hyperacetylation of histones.

SIRT1 demonstrates its anti-inflammatory effects in adipose tissue by preventing macrophage accumulation caused by high-fat feeding. It achieves this by repressing the expression of pro-inflammatory genes in adipocytes, potentially through deacetylating nuclear factor κB (NFκB) and inhibiting its binding to target gene promoters [42, 43]. In our investigation, we uncovered a substantial inverse relationship between *SIRT1* gene expression and levels of TNF-α levels, suggesting a potential role for SIRT1 in governing inflammation. Mariani et al. also examined the association between SIRT1 circulating level and some inflammatory factors, including ESR, CRP, and fibrinogen. Their findings revealed a negative correlation between SIRT1 and ESR, as well as fibrinogen [33]. On the other hand, we found a significant positive correlation between histone acetylation levels and TNF-α, proposing histone hyperacetylation as a contributing factor in the low-grade inflammation that is commonly observed in obesity. In line with our findings, Mikula et al. conducted an experimental animal study to explore the epigenetic changes associated with obesity at two critical inflammatory genes, TNF- α and Ccl2. They employed a high-fat diet-induced murine model of obesity and discovered a significant increase in TNF-α and Ccl2 mRNA levels in obese mice, which were tightly correlated with the histone H3 acetylation augmentation [44]. This finding aligns with our study, emphasizing the crucial role of inflammation in the context of obesity and highlighting the potential contribution of epigenetic mechanisms, specifically histone acetylation, in regulating the inflammatory response and its implications for the development and progression of obesity and its related complications like insulin resistance and MetS.

Other than indices of obesity, in this study there were significant differences in some of the anthropometric and biochemical parameters in the case and control groups, including SBP, DBP, HDL-C, FPG, insulin, and HOMA-IR, which could be attributed to the obesity. Unfavorable lipid levels and disturbed glycemic status have been previously reported to be relatively common among obese children [45, 46]. However, the difference between total cholesterol and HDL-C levels were not significant, which might be due to the young age of the studied subjects. Here we also found significantly higher height and height z-score in children with obesity. It has been previously reported that obesity is accompanied by increased linear growth, which might be due to the effect of insulin on IGF-1 receptor, and that children with obesity are taller for their age, although they do not usually tend to attain taller height as adults [47]. Nevertheless, no significant correlation was found between histone acetylation and height of the studied subjects.

The limitation of this study was that we focused on SIRT1 as the critical regulator of histone acetylation; however, other enzymes responsible for the acetylation-deacetylation of histones might also be related to obesity, which required complementary investigations, especially on larger study groups. Further comprehensive investigations are warranted to unravel the intricate interplay between the epigenetic modifications and obesity, ultimately fostering the development of targeted therapeutic interventions.

## Conclusion

In conclusion, the present study revealed the increased global histone acetylation in children and adolescents with obesity, in line with the downregulation of SIRT1 expression, and a remarkable association between histone hyperacetylation and insulin resistance. By elucidating the association between SIRT1 expression, histone acetylation, and obesity, our study highlights the significance of epigenetic modifications in obesity and its accompanying insulin resistance. These findings pave the way for further research exploring the modulation of SIRT1 and histone acetylation as potential therapeutic strategies for addressing the metabolic disturbances associated with obesity.

## Supporting information

S1 Data (SAV)
